# Wing Shape Variation between Terrestrial and Coastal Populations of the Invasive Box Tree Moth, *Cydalima perspectalis*, in Croatia

**DOI:** 10.3390/ani13193044

**Published:** 2023-09-28

**Authors:** Darija Lemic, Helena Viric Gasparic, Patricija Majcenic, Ivana Pajač Živković, Mario Bjeliš, Manuel J. Suazo, Margarita Correa, Jordan Hernández, Hugo A. Benítez

**Affiliations:** 1 Department of Agricultural Zoology, Faculty of Agriculture, University of Zagreb, Svetošimunska 25, 10000 Zagreb, Croatia; dlemic@agr.hr (D.L.); majcenic.pa3cija@gmail.com (P.M.); ipajac@agr.hr (I.P.Ž.); 2 Department of Marine Studies, University of Split, Ruđera Boškovića 31, 21000 Split, Croatia; mbjelis@unist.hr; 3 Instituto de Alta Investigación, Universidad de Tarapacá, Casilla 7D, Arica 1000000, Chile; suazo.mj@gmail.com; 4 Laboratorio de Ecología y Morfometría Evolutiva, Centro de Investigación de Estudios Avanzados del Maule, Universidad Católica del Maule, Talca 3466706, Chile; mcorreag@ucm.cl (M.C.); jordan.hernandez.01@alumnos.ucm.cl (J.H.); 5 Programa de Doctorado en Salud Ecosistémica, Centro de Investigación de Estudios Avanzados del Maule, Universidad Católica del Maule, Talca 3466706, Chile; 6 Cape Horn International Center (CHIC), Puerto Williams 6350000, Chile; 7 Centro de Investigación en Recursos Naturales y Sustentabilidad (CIRENYS), Universidad Bernardo O’Higgins, Avenida Viel 1497, Santiago 8370993, Chile

**Keywords:** box tree moth, geometric morphometrics, sexual dimorphism, invasiveness, wing shape

## Abstract

**Featured Application:**

**Use of Geometric morphometrics tools to understand invasiveness patterns of invasive species.**

**Simple Summary:**

The box tree moth is an invasive species that originated in Asia. Its presence causes damage by defoliating plants, to the point of causing their death. The presence of silk barriers and threads, covering plants during intense attacks, allows for species recognition. *Cydalima perspectalis* was first detected in 2007 in Germany and the Netherlands, after which it spread widely to other regions of Europe. In Croatia, its presence was first recorded in 2012 and it caused notable damage in 2013. This study analyzed the wing morphological variability of *C. perspectalis* in Croatia and its invasive character. This technique uses a mathematical approach in which the combination of geometry and statistics is essential to understand the morphology of invasive organisms; principally, how the wings adapt to allow the moth to colonize new environments. To achieve this, 269 moths from different areas were collected, and the wings of both males and females were evaluated. Significant differences in wing shape were found between terrestrial and coastal populations, with no sexual dimorphism established. The implications of this variability with regard to the invasive capacity and spread of the species are discussed.

**Abstract:**

The box tree moth (*Cydalima perspectalis* Walker, 1859; Lepidoptera: Crambidae) is an invasive species naturally distributed in Asia. The caterpillars in all developmental stages cause damage through defoliation of plants, and ultimately the death of the plant itself may occur. It is possible to recognize this species by its silk barriers and threads, and in the case of an intense attack, the entire plant will be covered with them. In Europe, this species’ presence was first recorded in 2007 in Germany and the Netherlands, and it is now widely distributed. In Croatia, its existence was first recorded in 2012, in Istria, while substantial damages were recorded in 2013. This work aimed to determine the morphological variability of *C. perspectalis* from Croatia and assess its invasive character, the possibility of flight, and the risk of further spread. The methods of geometric morphometrics were used as the analysis of wing shape. A total of 269 moths from different locations in Croatia were collected, the upper wings of males and females were analyzed using 14 landmarks. Significant differences in wing shapes between terrestrial and coastal populations were found, as well as subtle wing shape sexual dimorphism. The implications of this variability in species invasiveness and capacity of spread are discussed in this paper. We also extrapolate the usefulness of our results and suggest strategies for predicting and managing invasive species.

## 1. Introduction

Invasive insect species pose a significant threat to both agriculture and biodiversity, and their proliferation is on the rise due to the escalating global trade [1]. The surge in international trade has been identified as a primary driver of insect invasions across the world, as insects often act as hitchhikers on different trade goods such as plants, cereals, wood, etc. [2,3,4]. For western European countries, the liberalization of commercial policies within the European Union (EU) has further facilitated the trade of ornamental plants, potentially contributing to the dissemination of invasive insects [5]. The small size of insects in their immature stages contributes to the dissemination of these kinds of invaders. Understanding the pathways through which insects infiltrate different geographical regions is crucial for pinpointing vulnerabilities in international trade and devising classical biological control strategies and, last but not the least, strict trade policies [6]. It is equally important to comprehend the subtle shifts in insect populations that fuel their invasive behavior, whether they are from a genetic, phenotypic or ecological source [7]. Moreover, it was proved that invasive populations outside their origin area often suffer a change and become the source of invasive individuals [8]. Invaded countries often suffer multiple introduction events before the presence of the invasive insects is detected; these invasive populations, usually with different origins, can suffer admixture processes, thus becoming an invasive population [9].

The box tree moth (*Cydalima perspectalis* Walker, 1859; Lepidoptera: Crambidae), an invasive species originally from East Asia [10], exemplifies the impact of biological invasions on economic activities and nature. Indigenous to China, Korea, and Japan, *C. perspectalis* primarily feeds on species of the genus *Buxus*, which are commonly utilized as ornamental plants across the globe. The rampant trade of ornamental plants has been identified as a key factor responsible for the species’ invasive spread in Europe [11]. Possessing rapid propagation rates and strong adaptability, *C. perspectalis* made its European debut in 2007 in southwestern Germany and the Netherlands [12]. The inadvertent introduction of *C. perspectalis* to Europe is presumed to have occurred via infested box tree seedlings from China [13]. Over the past decade, the pest has rapidly disseminated across the European continent, posing a significant threat to native *Buxus* genus plants. In Croatia, the first sighting of *C. perspectalis* occurred in 2012 in the Istria region near the Croatian–Slovenian border [14].

The destructive impact of this insect primarily hinges on its larval stage, also known as caterpillars. These caterpillars inflict substantial damage by defoliating plants. A single caterpillar can consume over 45 box leaves during its development, and box bushes can host several hundred of these caterpillars [15]. In addition to leaf consumption, they gnaw at the plant bark, disrupting water and nutrient flow and leading to plant decay [16]. The pest’s development cycle encompasses two to five generations annually, contingent on climate conditions [17,18].

Given the economic and ecological significance of *C. perspectalis* as an invasive species, previous research has predominantly focused on its biology, ecology, and harmful impact. However, a comprehensive analysis of its flight capabilities and possible adaptations once present in invaded territories of invasive *C. perspectalis* populations, which is crucial for predicting future invasions, has been notably lacking. The presence of “bridgehead populations”, as populations from an invaded area become the source of new invasions, has been previously documented for this species, with European countries becoming a source of secondary invasions [11,19]. Even if these new invasive populations mainly originate from major ornamental trade countries, whether other invaded countries, such as Croatia, could be a bridgehead populations source has yet to be determined.

Geometric morphometrics, a technique for analyzing wing shapes in invasive insects [20], has yet to be utilized in *C. perspectalis* research. Emerging in the late 1930s, morphometrics quantitatively measures and analyzes morphological parameters, aiding in species identification and assessing variations within species. While traditional morphometrics measures length, height, and depth, it lacks detailed shape insights. Scientists developed alternative methods to study morphological shape, including geometry, leading to techniques such as analyzing external contours and specific points [21,22,23]. 

The core of the GM analysis is the combination of powerful multivariate statistics applied to the geometric information of the structures [22,24]. Studies of morphological diversification in invasive species have been performed principally on abdominal and wing morphology, and most of them have focused on reproduction associated with ecological preferences [25,26,27,28,29]. Geometric morphometrics offers advantages over traditional methods and is now a standard in many scientific research studies dealing with phenotypic variation [30,31,32]. This technique assesses the shape and size of morphological entities through mathematical forms and geometric principles [21], utilizing homologous specific points (landmarks) to detect shape changes linked to size variations [32,33]. This study employs geometric morphometrics to better understand the *C. perspectalis* wing shapes, providing valuable insights into this species’ invasive behaviors. When examining morphological adaptation, it is crucial to take into account the impact of allometry, which is defined as the relationship between an organism’s size and its shape or the co-variation of body parts due to changes in size [34,35]. Allometry can act as a developmental constraint [36,37], limiting the range of phenotypic variations that can arise from an organism’s developmental architecture. Although developmental constraints themselves may have arisen as a result of selective processes, they can still restrict the potential directions of phenotypic evolution [29,37,38].

Given the significance of *C. perspectalis* as both an insect pest and a biological invader, the primary objective of this study was to examine the morphological variability of *C. perspectalis* populations invading Croatia. This assessment aimed to ascertain its invasive nature, offering insights that could contribute to predicting potential flight capabilities, possible in situ morphological variation, and subsequent spread. 

## 2. Materials and Methods

### 2.1. Sampling and Identification

*Cydalima perspectalis* individuals were sampled at seven locations from the Republic of Croatia, where the insect has been previously detected. The identification was focused on the identification of collected adults and their wing patterns according to Mally and Nuss [13] (not larvae or pupae). In the case of field collection of preimaginal stages of the box plant (larvae and/or pupae), specimens were transferred to the laboratory to complete their development into pupae, and after maturation of the pupae we collected adults and performed identification based on their wing patterns.

**Figure 1:**
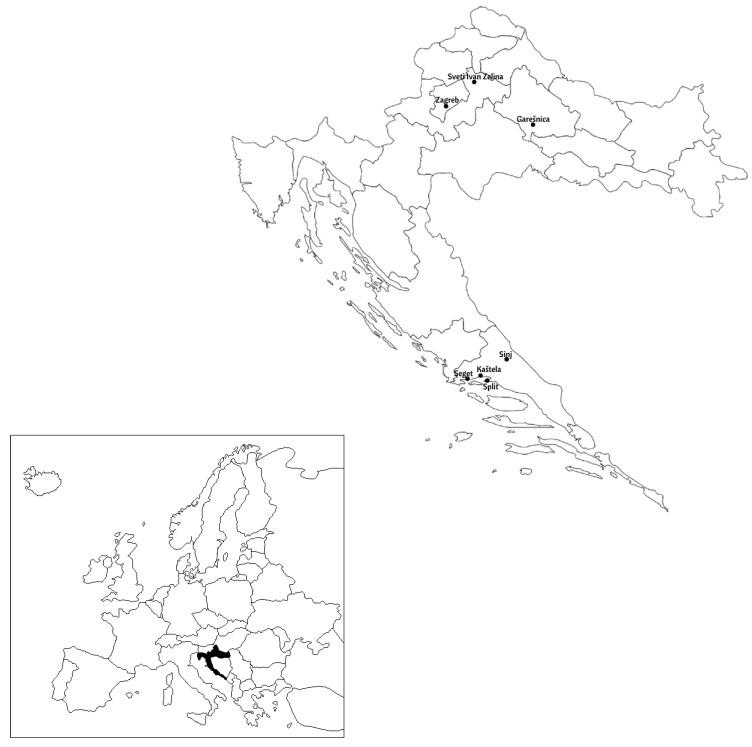
Geographical map of Croatia with locations from which terrestrial and coastal populations of *C. perspectalis* were collected.

Populations of *C. perspectalis* were separated into two groups according to their origin (coastal or terrestrial locations). For the terrestrial locations, populations from Zagreb, Sveti Ivan Zelina and Garešnica were sampled (Figure 1). For coastal locations, the sampling locations were Split, Sinj, Seget and Kaštela (Figure 1).

The initial sample collection for research commenced in April and May of 2021, coinciding with the emergence of *C. perspectalis* caterpillars. Caterpillars in their final developmental stage, along with pupae of *C. perspectalis*, were manually collected and placed within hunting cages separately. These caterpillars were reared within the cages until they reached the adult stage. Furthermore, adult *C. perspectalis* were captured using pheromone traps during early May 2021. These traps were strategically positioned near boxwood plants at a height of 1.5–2 m above the ground to maximize the probability of capture. Every two weeks, the pheromone was replaced, allowing for the capture and retrieval of first-generation males.

Overall, a total of 269 *C. perspectalis* adults were collected from different locations in the Republic of Croatia (Table 1) with 131 females and 138 males.

### 2.2. Data Acquisition

After capturing adult *C. perspectalis*, we differentiated between sexes based on the distinctive male “comb” at the abdomen’s tip [39]. The wings were carefully separated from the body, then cleaned with chlorine for scale removal. Once transparent, each wing was meticulously positioned on an object slide, affixed with Euparal adhesive, and covered with slips. Each slide was labeled with sample details such as the population, sampling location, and date. 

### 2.3. Designation of Specific Landmarks

**Figure 2:**
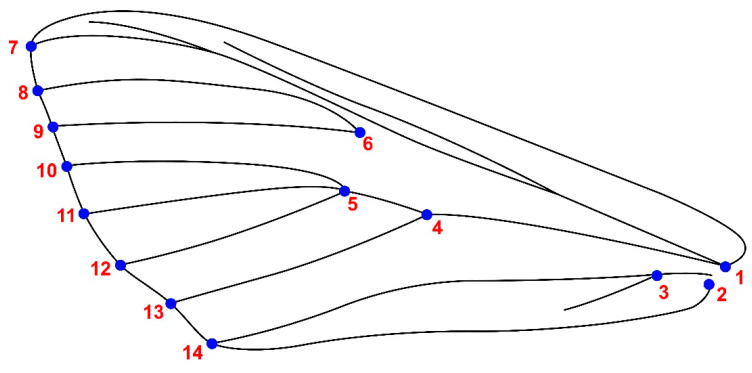
Upper wing of *Cydalima perspectalis* with 14 landmarks.

Upper wing images were captured with a Nikon D780 camera, Tokyo, Japan, ensuring individual sharpening and photography for each wing. These images were saved in jpg format and tagged with respective locations. Landmarking was accomplished using tpsDig2 v2.31 software, involving the strategic placement of fourteen landmarks (refer to Figure 2) at vein intersections and edges. Any samples featuring wing damage or overlapping were excluded from subsequent analyses. A comprehensive total of 412 wings (206 left and 206 right wings) underwent a successful preparation process, comprising 222 male (111 left and 111 right) and 190 female (85 left and 85 right) upper wings.

### 2.4. Data Analysis

Using MorphoJ 1.07a [40], the cartesian coordinates from the landmarks were processed via a generalized Procrustes analysis [41]. This method allows upper wing shapes to be compared by calculating an average configuration that encapsulates the selected reference points’ configurations. Principal Component Analysis (PCA) was used to explore the geometric morphospace, generating a scatter plot of the first two dimensions (PC) based on the covariance matrix of individuals included in the supplementary materials. For inter-locality comparisons, the mean shape covariance matrix extracted the average wing shape from each locality, followed by graphing and superimposing thin-plate spline transformations of the wing shape. To identify significant differences between population and sexual dimorphism, a multivariate analysis of variance (ANOVA) was performed. To assess the impact of size on shape (allometry), a multivariate regression was conducted, using wing shape as the independent variable and centroid size as the dependent variable [42]. Lastly, Canonical Variance Analysis (CVA) was employed to accentuate shifts in wing shape across different localities, capitalizing on CVA’s ability to maximize variation by generating new shape axes.

## 3. Results

The results presented herein demonstrate the effectiveness of geometric morphometrics in discerning wing shape distinctions among geographically diverse *C. perspectalis* specimens sourced from Croatian populations. The outcomes distinctly showcase discernible differences in wing shape between *C. perspectalis*. specimens from terrestrial (Sveti Ivan Zelina, Zagreb, Garešnica) and coastal (Split, Seget, Sinj, Kaštela) localities in Croatia. 

The wing shape variability is manifested through the first two dimensions of the morphospace, capturing a substantial 65.6% of the overall shape variance, specifically PC1: 52.08% and PC2: 12.98%. The significant variance in the initial component is not attributable to allometry, where multivariate shape regression holds marginal influence (prediction: 0.6%).

**Figure 3:**
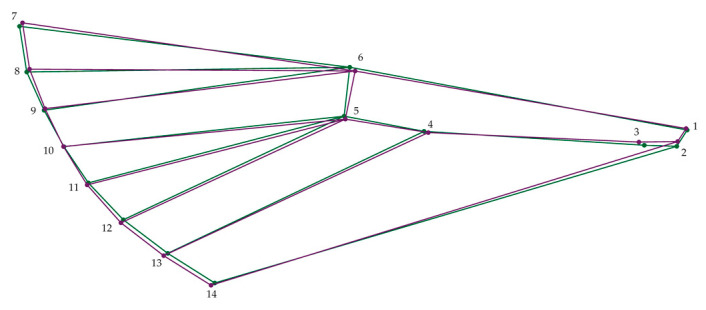
Wireframe representation variations in the average wing shape of box tree moth populations. Green: North, Purple: South.

The average shape PCA analysis also revealed that the wings of terrestrial *C. perspectalis* populations exhibit greater width in comparison to the wings of coastal populations. This expansion in wing width is particularly pronounced in the shifting of specific landmarks 4 and 6, wherein landmark 6 moves leftward and point 4 converges toward it. Furthermore, the findings indicate a slight elongation in the wings of coastal *C. perspectalis* populations in contrast with their terrestrial counterparts. This elongation primarily manifests through the displacement of specific points 2, 3, and point 6 to the right (Figure 3).

**Figure 4:**
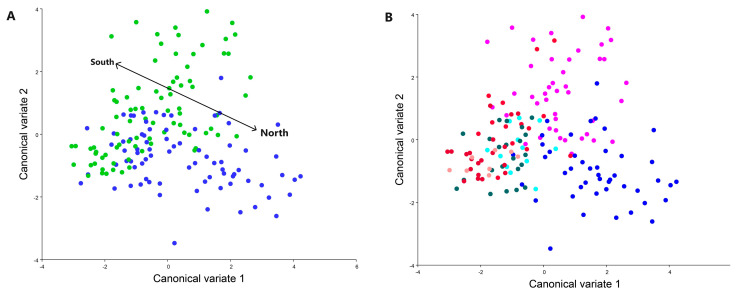
Canonical variate analysis between all populations studied, (**A**): The line divides two groups of northern (blue) and southern (green) populations. (**B**): This graph represents a blue tone to represent populations from the north Sveti Ivan Zelina, Garešnica and Zagreb, and from the red tone populations from the south Kaštela, Sinj, Split and Seget.

Upon conducting canonical variate analysis (CVA), clear distinctions emerged between the northern and southern populations of *C. perspectalis* within the Republic of Croatia (Figure 4). 

These results were confirmed via an ANOVA using the Procrustes coordinate between populations (ANOVA: F = 3.90 *p* ≤ 0.0001). Within terrestrial populations, noticeable distinctions were identified among *C. perspectalis* populations in the Zagreb and Sveti Ivan Zelina regions, in comparison to the Garešnica population. A permutation test using the Mahalanobis distances between all populations was performed to compare all groups to each other; only a few populations were found to have no significant differentiation. (Table 2).

**Figure 5:**
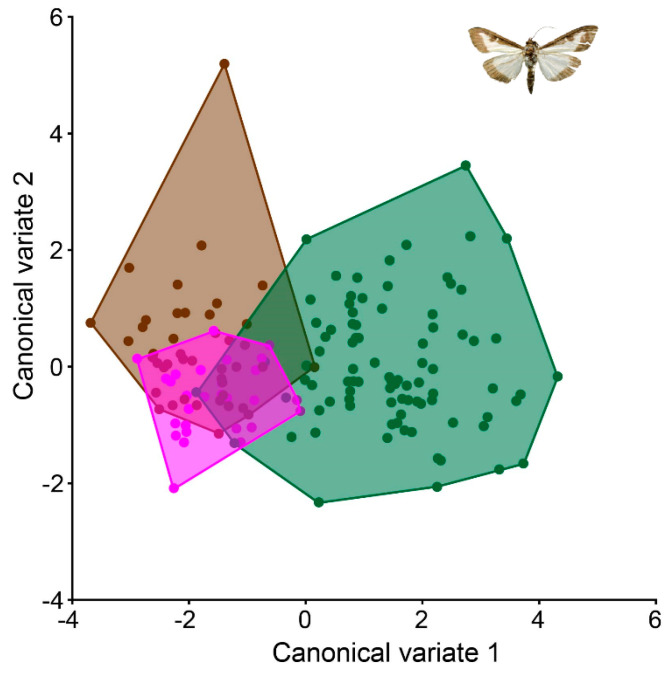
Canonical variate analysis between north terrestrial populations. Pink: Sveti Ivan Zelina populations; green: Garešnica population; and brown: Zagreb population.

**Figure 6:**
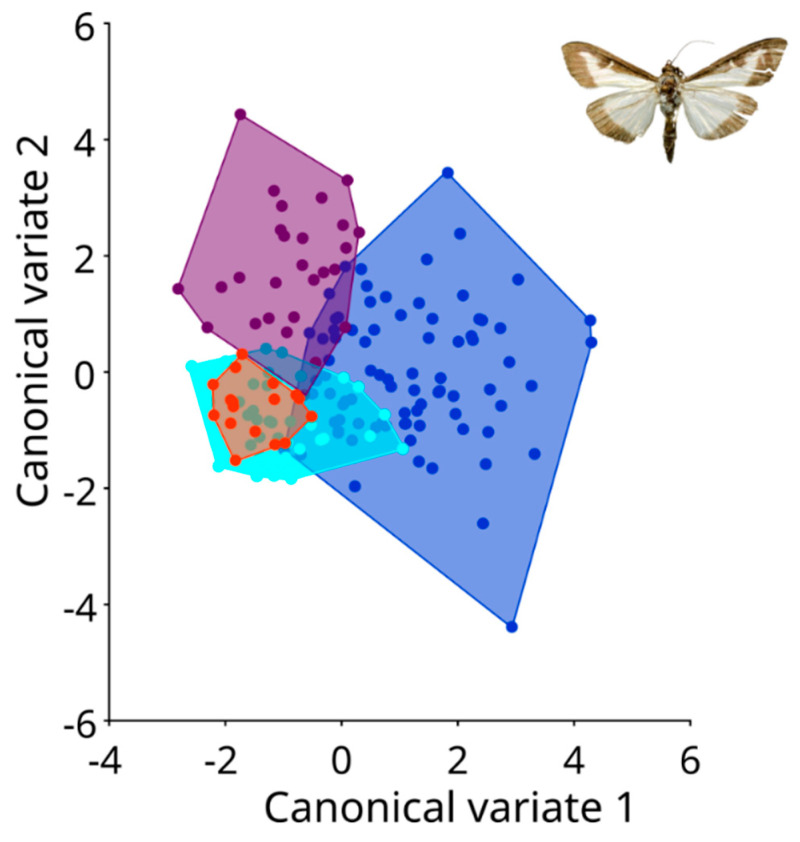
Canonical variate analysis between coastal populations. Blue: Kaštela population; light blue: Sinj population; purple: Split population; and orange: Seget population.

The wing shapes of *C. perspectalis* in the Zagreb and Sveti Ivan Zelina areas exhibited greater similarity (Figure 5). Conversely, in coastal populations of *C. perspectalis*, a considerable level of intrapopulation variability was observed. Particularly striking is the marked wing shape disparity between moth populations in Split versus those in Seget and Sinj. Similarly, while box tree moth populations from the Kaštela region displayed uniform wing shapes within specimens, distinct differences emerged when compared to the aforementioned Split, Sinj, and Seget populations, underscoring discernible wing variations (Figure 6).

**Figure 7:**
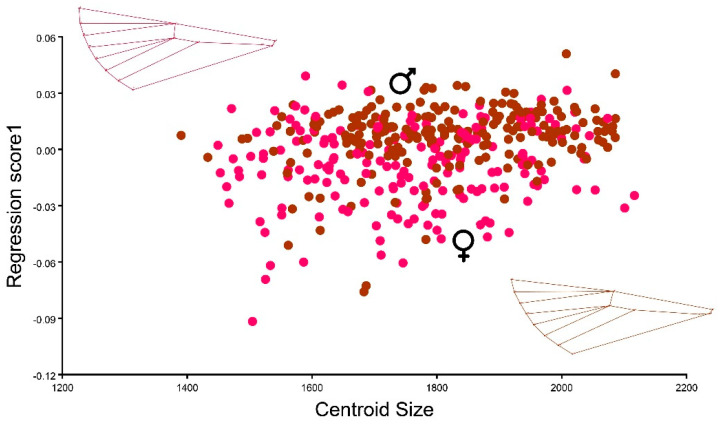
Multivariate regression of the *C. perspectalis* between centroid size (independent variable) and shape (dependent variable) of males (brown) and females (red).

Regarding sexual dimorphism, a multivariate regression analyzing wing shape in relation to centroid size indicated subtle distinctions between the wings of male and female *C. perspectalis*. While wing shape exhibited variability within the female population, it displayed limited variation among males (ANOVA: F = 19.84 *p* ≤ 0.0001). Therefore, a subtle sexual dimorphism was found between the observed populations (Figure 7).

## 4. Discussion

This study has introduced a novel dimension to the analysis of *Cydalima perspectalis*, providing illuminating insights into its potential for future invasions. The main objective of this research was to unravel the intricate morphological variations among *C. perspectalis* populations within Croatia, with specific emphasis on its invasive attributes, flight capabilities, and potential for further dispersion. 

Through rigorous geometric morphometric analysis, several compelling findings have come to light:

1. Distinct Geographical Wing Shape Disparities: Our research convincingly establishes significant differences in wing shapes between terrestrial (Zagreb, Sveti Ivan Zelina, Garešnica) and coastal (Kaštela, Seget, Split, Sinj) populations of *C. perspectalis*. This underscores the pivotal role of wing morphology in influencing the species’ spread.
2. Intraspecific Variation: Within terrestrial populations, notable differences were observed between Garešnica, Zagreb, and Sveti Ivan Zelina. Similarly, discernible wing shape variations were evident among coastal populations, including Split, Seget, Sinj, and Kaštela. These nuanced findings underscore the complexity of the species’ adaptation and dispersal mechanisms.
3. Exploring Sexual Dimorphism: Despite marked variability in the upper wings of male and female *C. perspectalis* individuals, our study does not definitively establish sexual dimorphism in wing shape. This suggests the potential involvement of other factors driving the observed variations.

Insect wings have emerged as pivotal structures for morphometric research due to their inherent two-dimensionality and transparency [27,43,44]. This unique wing quality has enabled researchers to delve into essential aspects of insect biology, such as determining population structure in cases where genetic markers may be insufficient [45,46]. Understanding population dynamics is crucial for devising effective pest control strategies [47,48].

Geometric morphometrics, with its precision in detecting subtle shape changes, offers a distinct advantage over genetic markers in elucidating population structure. The expedited detection of genetic changes through phenotypic alterations, as opposed to gradual changes in genotypic characteristics, provides a practical edge when it comes to monitoring and assessing population dynamics [45]. In the realm of entomology, the exploration of sexual dimorphism assumes significance, especially when sex differences may be inconspicuous due to species-specific traits or small physical sizes. Geometrically defining key anatomical coordinates enhances sex determination accuracy [26], which contributes to understanding sexual indices with implications for reproductive potential. The overarching phenomenon of larger females closely tied to abdominal expansion during egg development and offspring care [49,50,51] plays a significant role in shaping population dynamics. Variances from this norm, such as larger males due to intersexual competition [52], further underscore the intriguing complexities of insect biology. In some taxa like butterflies, differences in body and wing coloration underscore the multifaceted nature of sexual dimorphism [53].

No difference between female and male wings was detected. The absence of sexual dimorphism in wing shape, despite the established efficacy of geometric morphometrics in detecting various entomological phenomena, including sexual dimorphism [54,55,56,57], highlights the intricate interplay of factors contributing to the observed variations in *C. perspectalis* populations. Despite the lack of sexual dimorphism in wing shape, our study aligns with growing research that explores the intricate relationships between morphology, genetics, and population dynamics. This deeper understanding not only unveils the invasive tendencies of *C. perspectalis* but also contributes to a more nuanced comprehension of its ecological role and potential management strategies.

In the context of a species’ adaptability to new environments, it is vital to recognize the role of environmental influences [47]. The observed variability in wing shape among terrestrial and coastal populations highlights the potential impact of agroecological factors on morphological traits, which, in turn, can significantly influence a species’ capacity to expand into novel territories. The distinctions in wing shape between these populations may hold implications for future invasions of *C. perspectalis*. The broader wings seen in terrestrial populations may suggest enhanced maneuverability and extended flight capabilities, possibly facilitating rapid colonization. In contrast, the elongated wings observed in coastal populations may indicate a strategy for covering greater distances during dispersal. Such elongated wings are recognized for their aerodynamic advantages and are essential to the migratory movements of insects, as seen in the case of the western corn rootworm [48]. Furthermore, Mikac et al. [49] have proposed that these phenotypic differences in wing shape and size bear significant implications for the dispersal of both resistant and nonresistant insects, as wing morphology plays a pivotal role in an insect’s dispersal ability [50]. Notably, a study by Pajač Živković et al. [51] was the first to establish significant differences in wing shape among lepidopterans concerning resistance, emphasizing the need for careful consideration of resistance development in the future of *C. perspectalis*.

Many studies have explored the potential of using wing shape as an indicator of the environmental conditions of (*Aedes albopictus* and *Aedes aegypti*) mosquitoes [58]. While it was found that wing shape was not a reliable indicator of these conditions, this underscores the importance of understanding factors influencing flight and dispersal capabilities. Geometric morphometrics offers valuable insights into biology, particularly when distinguishing within and among species, sexual dimorphism, and other factors [58]. These techniques are especially relevant for epidemiologically significant insects, providing tools for enhanced species identification and insights into their invasiveness and ecology [58]. Additionally, research on *Anopheles superpictus* highlights the impact of temperature on morphology and development, shedding light on their flight capabilities and longevity, which is crucial for understanding disease transmission dynamics [58].

Furthermore, the concept of phenotypic plasticity, where a species responds to environmental changes [58] aligns well with our findings. *Cydalima perspectalis*’ adaptability to diverse agroecological conditions, resulting in variations in wing morphology, may enhance its capacity for successful invasions. The detected but low morphological variability between populations from apparently very different agroecological systems that was investigated in this study indicates the stability of the *C. perspectalis* genotype, which is reflected in a stable phenotype (explained in Bouyer et al. [33]).

Importantly, these insights hold substantial implications for future pest management strategies, especially considering the current lack of comprehensive control measures for *C. perspectalis* [59]. As this species continues its invasive trajectory, understanding the interplay between its wing morphology and adaptability will be crucial for designing effective biosecurity measures to curb its proliferation in new environments. The role of the ornamental plant trade has played an important role in biological invasions [60]; this trade is also responsible for the *C. perspectalis* invasion through Europe. Considering the high prevalence and invasiveness of *C. perspectalis*, as well as its proven ability to adapt to different agroecological conditions (phenotypic plasticity), it is expected that the *C. perspectalis* will spread and adapt to previously uninfected areas during periods of significant climatic change. The use of mathematical modeling by using the widely available existing data, such as those produced by this study, to anticipate biological invasions [61] to generate public policies should be a priority to prevent new situations such as the one created by *C. perspectalis*.

## 5. Conclusions

This study has provided valuable insights into the morphological variability of *Cydalima perspectalis* populations within Croatia, shedding light on its invasive tendencies, flight capabilities, and adaptability to diverse agroecological conditions, such as the ones studied here (coastal and terrestrial). Even though the biological invasion of *C. perspectalis* in Europe has become an inevitable reality, we were able to select key traits to study in invasive Lepidoptera species. These results can also be used to nurture mathematical models’ parameters to predict and anticipate invasions in a climate change frame.

Through the application of geometric morphometric methods, we have identified significant differences in wing shapes between terrestrial and coastal populations, underscoring the potential role of wing morphology in the species’ spread. Despite the absence of sexual dimorphism in wing shape, our findings contribute to the broader understanding of the intricate interplay between morphology, genetics, and population dynamics. As *C. perspectalis* continues its expansion, these findings hold implications for designing effective biosecurity measures and informed pest management strategies, ultimately helping to curtail the proliferation of this invasive pest in new environments.

## Figures and Tables

**Table 1:** Details of the samples used in this study, sampling method and number of moths obtained for the study.

Location	Population	*n*	Females	Males	
			Hand Collection	Hand Collection	Pheromone Trap
Terrestrial	Sveti Ivan Zelina	20			20
	Zagreb	78	22	56	
	Garešnica	47	28	19	
Coastal	Seget	10	5	5	
	Split	19	18	1	
	Sinj	33	17	16	
	Kaštela	62	41	21	

**Table 2:** Permutation test between northern and southern populations using the Mahalanobis distances, *p*-values from permutation tests (10,000 permutation rounds). North populations: Sveti Ivan Zelina, Garešnica and Zagreb. South Populations: Kaštela, Sinj, Split, and Seget.

Locality/*p*-value	Sveti Ivan Zelina	Seget	Garešnica	Zagreb	Kaštela	Sinj
Seget	2.168 0.0471					
Garešnica	2.7558 <0.0001	3.7601 <0.0001				
Zagreb	1.3877 0.6057	1.8757 0.0727	3.0898 <0.0001			
Kaštela	2.5234 0.0015	3.534 <0.0001	2.8234 <0.0001	2.8728 <0.0001		
Sinj	1.7339 0.0502	1.4956 0.2306	3.7198 <0.0001	1.3753 0.0922	3.264 <0.0001	
Split	2.2419 0.0436	2.9868 0.0001	3.4462 <0.0001	2.4283 <0.0001	2.6359 <0.0001	2.705 <0.0001

## Data Availability

Data can be made available by personal request to the corresponding author.

## Supplementary Materials

The following supporting information can be downloaded at: https://www.mdpi.com/article/10.3390/ani13193044/s1, Text S1: covariance matrix.
